# Using High Frequency Transcranial Random Noise Stimulation to Modulate Face Memory Performance in Younger and Older Adults: Lessons Learnt From Mixed Findings

**DOI:** 10.3389/fnins.2018.00863

**Published:** 2018-11-29

**Authors:** Tegan Penton, Sarah Bate, Kirsten A. Dalrymple, Thomas Reed, Maria Kelly, Sheina Godovich, Marin Tamm, Bradley Duchaine, Michael J. Banissy

**Affiliations:** ^1^MRC Social, Genetic and Developmental Psychiatry Centre, Institute of Psychiatry, Psychology and Neuroscience, King’s College London, University of London, London, United Kingdom; ^2^Department of Psychology, Bournemouth University, Poole, United Kingdom; ^3^Institute of Child Development, University of Minnesota, Minneapolis, MN, United States; ^4^Department of Experimental Psychology, University of Oxford, Oxford, United Kingdom; ^5^Department of Psychology, Goldsmiths, University of London, London, United Kingdom; ^6^Department of Psychological and Brain Sciences, Dartmouth College, Hanover, NH, United States

**Keywords:** transcranial random noise stimulation, transcranial electrical stimulation, individual differences, face memory, face recognition, aging

## Abstract

High-frequency transcranial random noise stimulation (tRNS) has been shown to improve a range of cognitive and perceptual abilities. Here we sought to examine the effects of a single session of tRNS targeted at the ventrolateral prefrontal cortices (VLPFC) on face memory in younger and older adults. To do so, we conducted three experiments. In Experiment 1, we found that younger adults receiving active tRNS outperformed those receiving sham stimulation (i.e., using a between-participant factor for stimulation condition; Experiment 1). This effect was not observed for object memory (car memory) in younger adults (Experiment 2), indicating that the effect is not a general memory effect. In Experiment 3, we sought to replicate the effects of Experiment 1 using a different design (within-participant factor of stimulation – active or sham tRNS to the same individual) and to extend the study by including older adult participants. In contrast to Experiment 1, we found that active tRNS relative to sham tRNS reduced face memory performance in both younger and older adults. We also found that the degree of decline in performance in the active tRNS relative to sham tRNS condition was predicted by baseline ability, with higher performing participants showing the largest decreases in performance. Overall, the results indicate that tRNS to the VLPFC modulates face memory, but that there may be performance and protocol specific moderators of this effect. We discuss these findings in the context of the broader literature showing the importance of individual variation in the outcome of non-invasive brain stimulation intervention approaches. We conclude that while tRNS may have potential as an intervention approach, generalizing from single experiment studies to wide application is risky and caution should be adopted in interpreting findings.

## Introduction

The ability to recognize a face is an important skill that depends on a variety of cognitive and neural processes (Bruce and Young, 1986; Haxby et al., 2000; Duchaine and Yovel, 2015). Face recognition ability has been suggested to peak in adulthood around the age of 30–34 years old with a steady decline in these skills observed after this period (Germine et al., 2011). Failure to develop normal face recognition abilities has been linked with negative psychosocial consequences (e.g., Yardley et al., 2008; Dalrymple et al., 2014a). Conversely, some people show extraordinarily good face recognition (Russell et al., 2009; Bobak et al., 2016) and these skills are valuable in a number of professions (White et al., 2014). Potential methods to improve face recognition abilities therefore warrant investigation, as they may ameliorate face-processing deficits in atypical groups or facilitate face processing in typical adults across the lifespan.

Recent evidence demonstrates that non-invasive brain stimulation can improve a range of cognitive and perceptual abilities. For instance, Snowball et al. (2013) report that pairing multiple sessions of high-frequency transcranial random noise stimulation (tRNS) with cognitive training can enhance arithmetic learning, with the benefits persisting six months after training. During tRNS a weak current is passed between two electrodes placed on the scalp. In high-frequency tRNS, an alternating current ranging randomly between 100 and 640 Hz is administered. This has been shown to lead to an increase in excitation under both scalp electrodes (Terney et al., 2008). While the mechanisms of action are not well understood, one suggestion is that mechanisms of stochastic resonance contribute to the effect with random noise amplifying weak neural signals (e.g., Moss et al., 2004).

Transcranial random noise stimulation has also been used to investigate aspects of social perception and cognition. For example, in our prior work, we have shown that tRNS to bilateral occipitotemporal cortices can enhance facial identity perception, but not facial trustworthiness perception, in typical younger adults. This effect was not observed following either sham or sensorimotor cortex stimulation (Romanska et al., 2015). In other work, alternative forms of transcranial electrical current stimulation have also been shown to modulate aspects of memory for faces in younger adults (Lafontaine et al., 2013; Renzi et al., 2015; Barbieri et al., 2016). For instance, Barbieri et al. (2016) demonstrated that offline anodal transcranial direct current stimulation (a-tDCS; a form of transcranial electrical current stimulation using a direct current to increase cortical excitation to the area of cortex underneath a single electrode) over the right occipital cortex enhanced face and object memory in typical younger adults. These findings suggest non-invasive brain stimulation is a promising tool to enhance facial identity processing skills in typical younger adults, but the extent to which face memory can be modulated with tRNS and whether any beneficial effect of stimulation extends to other groups (e.g., older rather than younger adults) remains unknown.

Here, we conducted three experiments to examine the impact of a single session of high-frequency tRNS targeted at bilateral ventrolateral prefrontal cortices (VLPFC) on face (Experiments 1 and 3) and object (Experiment 2) memory in younger adults, and face memory in older adults (Experiment 3) adults. Our selection of VLPFC was based on neuroimaging and brain stimulation work showing: increased fronto-central activation (including bilateral inferior frontal gyri) when making correct rejections of distractor faces during a face memory task (Hofer et al., 2007), contributions of the VLPFC to facial identity processing (Grady et al., 2002; Fairhall and Ishai, 2007; Thomas et al., 2008; Oh and Leung, 2010; Guntupalli et al., 2016), evidence of face-selective lateral prefrontal cortex activation (Chan and Downing, 2011; Chan, 2013), and demonstration that intracranial stimulation of the right inferior frontal gyrus (part of the VLPFC) elicits face percepts (Vignal et al., 2000). Given these findings, we predicted that tRNS to VLPFC would improve face memory abilities.

## Experiment 1: Face Memory in Younger Adults

### Experiment 1 Materials and Methods

#### Participants

In Experiment 1, 28 Caucasian right-handed adults participated for a small monetary reward. Participants were randomly assigned to the active high-frequency tRNS group (*N* = 14; Mean Age = 29.6 years, SD Age = 10.1 years; 9 females) or the sham group (*N* = 14; Mean Age = 24.9 years, SD Age = 8.2 years; 8 females). The groups did not differ significantly in age [*t*(26) = 1.33, *p* = 0.194].

All participants were healthy volunteers, without any known developmental or neurological disorders and no contraindications to tRNS. They were naive with respect to the experimental hypothesis and remained unaware of what type of stimulation they received until the end of the experiment.

#### Brain Stimulation Parameters

High frequency tRNS was administered using a NeuroConn DC Plus Stimulator. Two 5 × 5 cm electrodes placed in saline soaked sponges were used. Stimulation was administered at 1.5 mA for 20 min, with a 15 s fade-in and fade-out time. An identical setup was used for the sham group, but stimulation was only administered for the first 30 s. This evokes the sensation of being stimulated, but does not lead to a neurophysiological change that can influence performance (Ambrus et al., 2010). The sites of stimulation were identified using the electroencephalography 10–20 system, with electrodes placed over the F7/F8 scalp electrode sites. Site selection was based on frameless stereotactic image guidance of scalp electrodes indicating that the F7/F8 electrodes are over the inferior frontal gyri, which form part of the VLPFC. Given the size of the electrodes used, the stimulation was also likely to extend laterally to include stimulation of neighboring regions that form part of the VLPFC including pars triangularis of the inferior frontal gyrus and the lateral surface of the ventral frontal cortex, thus we refer to the stimulated region as VLPFC rather than inferior frontal gyrus.

#### Materials and Procedure

Prior to the study, all participants were provided with written information about the study and a description of the tRNS procedure. The associated safety risks/warnings were explained, and participants were asked to sign an informed consent form. This study received full ethical approval by the local ethics committee.

**FIGURE 1:**
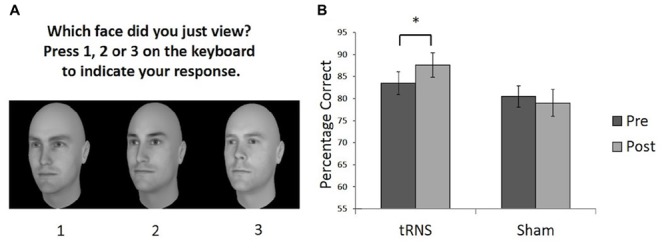
**(A)** Example of a typical test trial on the CFMT following the learning phase. **(B)** Performance on the CFMT prior to (pre) and following (post) brain stimulation. Active tRNS to VLFPC improved performance on CFMT in young adults. ^∗^*p* < 0.05. Error bars represent Standard Error.

To examine pre- versus post-performance in face memory, two versions of the Cambridge Face Memory Test (CFMT) were used (Bate et al., 2014). The CFMT is commonly utilized measure to study face memory (Duchaine and Nakayama, 2006a), where participants memorize six target faces and are asked to choose which one of three faces was a target. Responses were recorded via keypress. We used two versions of the CFMT with different faces, generated by FaceGen software for pre- and post-testing (Figure 1A). The use of the two versions of the CFMT rather than two administrations of the same test allows us to compare performance on measures matched for difficulty and task demands, while reducing the practice effects often present in a pre- versus post-performance design. A previous study demonstrated the tasks do not differ in difficulty and the order in which the tasks are completed does not influence performance (Bate et al., 2014). Each task requires learning and recognizing six unfamiliar male Caucasian faces in different views (left 1/3 profile, frontal, and right 1/3 profile) and lighting. Participants are then tested on their ability to recognize these faces in a three-alternative forced-choice task. A total of 72 trials are completed per test. The percentage of correct responses was measured. Feedback was not provided during the test.

The timeline for each testing session involved participants completing a pre-test CFMT. Next they received active or sham tRNS prior to starting the post-stimulation CFMT. In the active tRNS condition, participants received 20 min of stimulation, with 10 min of stimulation taking place prior to the task, and the remaining stimulation delivered while participants completed the post-stimulation task. In the sham condition, this procedure was mimicked. The two versions of the CFMT were counterbalanced across participants as the baseline or post-stimulation task (Figure 1A). The testing session took approximately 45 min to complete.

### Experiment 1 Results and Discussion

One participant in the stimulation group showed a difference score between pre- and post-stimulation that was greater than two standard deviations above the mean difference score for the group (i.e., they were an outlier by showing a greater improvement than the rest of the group) and was withdrawn from our analysis. To examine whether a single session of active high frequency tRNS facilitated face memory relative to sham stimulation a 2 (Stimulation Group) × 2 (Session) mixed ANOVA was conducted on the remaining participants (Sham group *N* = 14; tRNS group *N* = 13). This revealed a significant interaction between Stimulation Group and Session [*F*(1,25) = 5.73, *p* = 0.024, $\eta_{p}^{2}$ = 0.187]. Planned paired comparisons revealed the active tRNS group showed a significant improvement in performance after stimulation relative to baseline [*t*(12) = 3.43, *p* = 0.005, *r* = 0.25], but this pattern was not observed for the sham stimulation group [*t*(13) = 0.759, *p* = 0.462, *r* = -0.10] (Figure 1B). Further, the two groups did not differ significantly in performance before stimulation [*t*(25) = 0.758, *p* = 0.456, *r* = 0.15], but did significantly differ post stimulation [*t*(25) = 2.09, *p* = 0.047, *r* = 0.39]. The main effect of Group and Session were not significant [For Group: *F*(1,25) = 2.42, *p* = 0.133, $\eta_{p}^{2}$ = 0.088; For Session: *F*(1,25) = 1.27, *p* = 0.271, $\eta_{p}^{2}$ = 0.048].

These findings therefore indicate that a single session of active high frequency tRNS to VLPFC resulted in an enhancement in face memory performance in young adults. A similar modulation effect was not observed following sham stimulation in a different group of participants. This provides support for the potential utility of high frequency tRNS to VLPFC as a means to improve face memory skills and provides further evidence that the VLPFC plays a role in the face recognition abilities of typical young adults. The findings are consistent with literature indicating the importance of the VLPFC in facial identity processing in younger adults (Vignal et al., 2000; Fairhall and Ishai, 2007; Thomas et al., 2008; Oh and Leung, 2010; Renzi et al., 2013; Guntupalli et al., 2016). We add to this literature by demonstrating that changing cortical excitability in the VLPFC can lead to a modulation of face memory performance in young adults. It has previously been shown that transcranial magnetic stimulation (TMS) of the right inferior frontal gyrus disrupts the ability to perceive differences in the spacing of facial features, but not featural processing (Renzi et al., 2013). This may be one potential mechanism that could account for the changes in performance observed here (e.g., by influencing the perceptual encoding of unfamiliar faces).

There, are however, some important limitations to Experiment 1. Firstly, the lack of a non-face memory task means that it is unclear whether the modulation in performance following high frequency tRNS is related to face recognition or affects visual recognition memory in general. Secondly, our use of a between-participant design introduces the risk that differences may relate to some form of individual difference between the groups that was not controlled for in the current study. To address these issues, we conducted two additional experiments. In Experiment 2, we assessed whether high frequency tRNS targeted at VLPFC results in a modulation of non-face visual recognition abilities relative to sham stimulation. In Experiment 3, we assessed how high frequency tRNS targeted at VLPFC influences face memory in young and older adult participants when stimulation session is a repeated-measures factor (i.e., the same participants complete sham and active stimulation sessions).

## Experiment 2: Non-Face Visual Memory in Young Adults

Although Experiment 1 demonstrated an improvement in face memory following active high frequency tRNS targeted at the VLPFC, it is unclear whether this modulation in performance following high frequency tRNS is specific to face recognition or affects visual recognition memory in general. To address this issue, a second experiment was conducted to examine whether high frequency tRNS targeted at VLPFC would result in a modulation of non-face visual recognition abilities relative to sham stimulation.

### Experiment 2 Materials and Methods

#### Participants

In Experiment 2, 38 right-handed young adults participated for a small monetary reward. Participants were randomly assigned to either the active high-frequency tRNS (*N* = 19; 24.4 years, *SD* = 6.3 years, 9 females) or sham group (*N* = 19; *M* = 26.8 years, *SD* = 6.0 years, 8 females). The groups did not differ significantly in age [*t*(36) = 1.21, *p* = 0.234].

All participants were healthy volunteers, without any known developmental or neurological disorders and no contraindications to tRNS. They were naive with respect to the experimental hypothesis and remained unaware of what type of stimulation they received until the end of the experiment.

#### Brain Stimulation Parameters

The stimulation parameters were identical to Experiment 1 (see section “Experiment 1 Materials and Methods”).

#### Materials and Procedure

Prior to the study, all participants were provided with written information about the study and a description of the tRNS procedure. The associated safety/risk warnings were explained, and participants were asked to sign an informed consent form. This study received full ethical approval by the local ethics committee.

**FIGURE 2:**
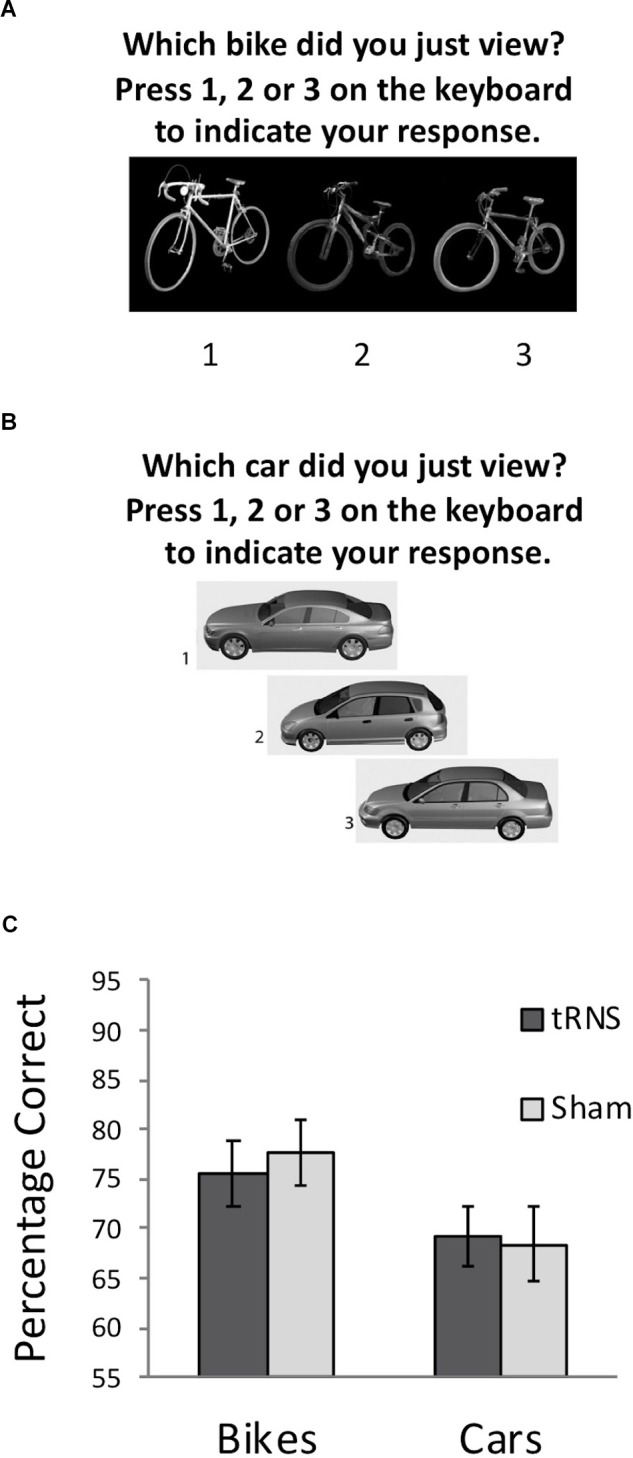
**(A)** Example of a typical test trial on the CBMT following the learning phase. **(B)** Example of a typical test trial on the CCMT following the learning phase. **(C)** Performance on the CBMT (prior to stimulation) and the CCMT (following stimulation) in both groups. No significant effect of active tRNS to VLPFC was observed. Error bars represent Standard Error.

Experiment 2 examined whether high frequency tRNS targeted at VLPFC would result in a modulation of non-face visual memory abilities. Two tasks were used to examine non-face memory abilities, both with the same format as the CFMT: Cambridge Bicycle Memory Test (CBMT) and the Cambridge Car Memory Test (CCMT). Our main task of interest was the CCMT (Dennett et al., 2012), which all participants completed following tRNS. In the CCMT, participants are shown three images of six different cars that vary in viewpoint: side-on, approximately 30° left from side-on, and approximately 30° right from side-on. As with the CFMT, participants are asked to learn the cars and are then tested with 72 trials on their ability to determine which one of three cars presented in each test item was a target (see Dennett et al., 2012 for full description). Thus, the task is matched in format to the CFMT, but only modestly correlates with the CFMT performance (Dennett et al., 2012; Shakeshaft and Plomin, 2015). The CBMT (Dalrymple et al., 2014b) also used the same format as the CFMT and CCMT, but involved participants learning and recognizing six different bicycles. The test was developed for use in children and thus is somewhat easier than the CCMT (which was designed for adults). In view of the differences in task difficulty between the CBMT and CCMT, the CBMT was used as a measure to ensure that the sham and active high-frequency tRNS group did not differ significantly in their baseline object recognition abilities. The timeline for each testing session involved all participants completing the CBMT at baseline as an index of participants’ baseline visual memory abilities (see Figure 2A). Following the CBMT, participants received 20 min of active or sham tRNS. As per Experiments 1 and 2, in the active tRNS condition, participants received 20 min of stimulation, thus 10 min of stimulation was conducted prior to the task, and the remaining stimulation was conducted while participants completed the CCMT (Figure 2B). The testing session took approximately 45 min to complete.

### Experiment 2 Results and Discussion

A 2 (Stimulation Group) × 2 (Task) mixed ANOVA was conducted. This revealed a significant main effect of Task [*F*(1,36) = 10.83, *p* = 0.002, $\eta_{p}^{2}$ = 0.231] driven by better performance on the bicycle task for all participants, which is due to differences in difficulty between the CCMT and CBMT noted earlier. No main effect of Stimulation Group was found [*F*(1,36) = 0.02, *p* = 0.894, $\eta_{p}^{2}$ = 0.001]. Importantly, the Task × Group interaction was also not significant [*F*(1,36) = 0.31, *p* = 0.580, $\eta_{p}^{2}$ = 0.009] indicating that high-frequency tRNS of VLPFC did not significantly modulate non-face visual recognition memory (Figure 2C).

To ensure that the slight, non-significant difference between the groups in the CBMT at baseline (Active *M* = 75.67, Sham *M* = 77.54; *t*(36) = -0.40, *p* = 0.691) did not influence the relationship between stimulation and CCMT performance, a secondary analysis of covariance was also run examining whether CCMT performance differed between groups when including CBMT performance as a covariate. No significant relationship was observed between stimulation condition and CCMT scores, *F*(1,35) = 0.17, *p* = 0.681, $\eta_{p}^{2}$ = 0.005). Collectively, these analyses suggest that high frequency tRNS to VLFPC does not lead to general improvements in visual memory.

## Experiment 3: Face Memory in Younger and Older Adults

The findings from Experiment 1 indicated that a single session of active high frequency tRNS to VLPFC (relative to sham stimulation) resulted in an enhancement in face memory performance for computer-generated faces when using a between-participants design. We conducted an additional experiment with different participants to examine whether we would find similar effects on face memory to Experiment 1 when using a repeated-measures approach (i.e., testing sham versus active stimulation in the same participants). This is an important consideration to ensure that a) prior effects replicate across different experimental designs, and b) that any prior results were not linked to any unforeseen individual differences between the groups.

In addition to introducing a repeated-measures factor for Stimulation Condition (active versus sham), we also wanted to consider an important between-participant factor that might influence the effect of stimulation – the age group of participants. There were two reason for doing this. Firstly, age has been shown to be an important individual difference factor that influence the outcome of brain stimulation interventions (e.g., see Krause and Cohen Kadosh, 2014; Fertonani and Miniussi, 2017). Secondly, aging has been related to declines in face memory performance - typically older adults show reduced face memory relative to younger adults (Lamont et al., 2005; Germine et al., 2011) - and we know that baseline performance is a common moderator of the outcome of brain stimulation interventions (e.g., see Krause and Cohen Kadosh, 2014; Fertonani and Miniussi, 2017). It is therefore important to consider how age influences the effects of high-frequency tRNS on face memory.

With this in mind, we investigated whether high frequency tRNS to VLPFC resulted in an enhancement in face memory using a mixed design with the factors of Session (sham versus active stimulation) and Age group (older versus younger adults).

### Experiment 3 Materials and Methods

#### Participants

In Experiment 3, 20 right-handed older adults (Mean Age = 67.2 years, SD Age = 6.4 years, 15 females) and 19 right-handed younger adults (Mean Age = 24.7 years, SD Age = 3.7 years, 15 females) participated for a small monetary reward. All participants were healthy volunteers, without any known developmental or neurological disorders and no contraindications to tRNS. All older adult participants were screened using the Mini Mental State Examination (MMSE). The MMSE is a commonly used measure to screen for cognitive status. A cut-off limit of <24 was used, which has a good sensitivity for dementia in the older population (Chayer, 2002). No participants were excluded from the study on the basis of this criterion.

#### Brain Stimulation Parameters

All participants received either active tRNS or sham stimulation in a counterbalanced order 1-week apart (i.e., a within-participant design was used). All other aspects of the stimulation protocol were identical to Experiment 1 (see section “Experiment 1 Materials and Methods”).

#### Materials and Procedure

Prior to the study, all participants were provided with written information about the study and a description of the tRNS procedure. The associated safety/risk warnings were explained, and participants were asked to sign an informed consent form. This study received full ethical approval by the local ethics committee.

A different version of the CFMT was completed in each of the two testing sessions. The versions used were the same as those used in Experiment 1 (see section “Experiment 1 Materials and Methods” for description). The timeline for each testing session involved participants receiving 20 min of active or sham tRNS (counterbalanced across testing sessions), with 10 min of stimulation taking place prior to the task, and the remaining stimulation conducted while participants completed the CFMT. There was a minimum of a 1-week gap between sessions to avoid carry-over effects. Each testing session took approximately 45 min to complete.

### Experiment 3 Results and Discussion

**FIGURE 3:**
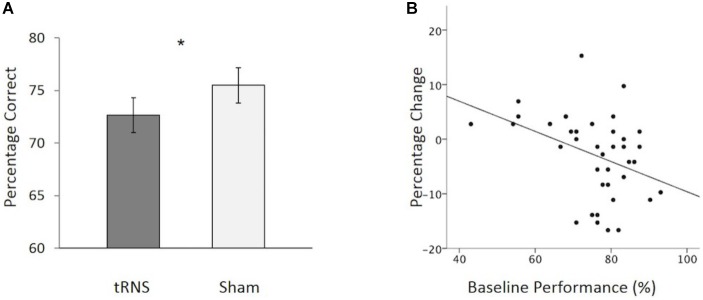
**(A)** Overall, participants showed a significant reduction in CFMT performance following active tRNS to VLPFC relative to sham. **(B)** Baseline performance predicts the degree of change following active tRNS to VLPFC with higher performing participants showing the greatest reduction in performance. ^∗^*p* < 0.05. Error bars represent Standard Error.

No outliers were observed in the data set. A 2 (Stimulation Condition) × 2 (Age Group) mixed ANOVA was conducted to examine if overall CFMT performance differed between active and sham tRNS as a function of age group. No significant interaction was found between Stimulation Condition and Age Group [*F*(1,37) = 0.052, *p* = 0.820, $\eta_{p}^{2}$ = 0.001]. There were, however, significant main effects of interest. Firstly, a main effect of Age Group was observed [*F*(1,37) = 9.226, *p* = 0.004, $\eta_{p}^{2}$ = 0.200], due to older adults performing worse than younger adults overall. Secondly, and contrary to Experiment 1, a main effect of Stimulation Condition was found [*F*(1,37) = 5.267, *p* = 0.028, $\eta_{p}^{2}$ = 0.125], which was due to participants performing worse in the active tRNS condition relative to the sham condition (Figure 3A). This finding of a group-level reduction in performance in active relative to sham tRNS conditions is inconsistent with the findings from Experiment 1 where active tRNS was found to aid performance relative to sham tRNS at the group level (Figure 1B).

To further explore this effect, we examined how individual differences in baseline performance (i.e., sham) performance were related to performance change following active stimulation. There were three reasons for doing this: (1) prior work has suggested that baseline ability can interact with stimulation outcome effects following transcranial electrical stimulation (tES) (e.g., Tseng et al., 2012; Hsu et al., 2014, 2016; Krause and Cohen Kadosh, 2014; Benwell et al., 2015; Fertonani and Miniussi, 2017; Penton et al., 2017; Tseng et al., 2018); (2) inter-individual differences in memory performance have been shown to increase with age (e.g., Morse, 1993); and (3) different patterns of activation of the VLPFC have been reported for high versus low- performing older adults (Cabeza et al., 2002). To explore possible individual differences, we conducted a hierarchal regression to examine how baseline performance influenced performance change following active stimulation (active tRNS performance minus sham tRNS performance). Participant age (years) and order of stimulation session testing (1 = active tRNS session first; 2 = sham session first) were entered into the first step of the model. No variables in the first step reached significance as predictors of performance change. Baseline performance was added in the second step. This resulted in a significant increase in the variance of performance change following stimulation that was accounted for [*F*(1,35) = 7.44, *p* = 0.01; 15.9% additional variance in performance change following stimulation explained]. Baseline performance acted as a significant predictor of the degree of change following active high frequency tRNS [β = -.468, *t* = -2.73, *p* = 0.010], but not age [β = -0.190, *t* = -1.11, *p* = 0.275] or order of stimulation session [β = -0.109, *t* = -0.72, *p* = 0.479]. In this regard, as baseline performance increased, performance (active tRNS relative to sham tRNS) decreased (Figure 3B). Collectively, these findings suggest that when using a repeated-measure design in younger and older adults, tRNS to VLPFC can lead to reductions in face memory performance. The degree of change following active tRNS relative to sham depends on baseline ability. This contrasts with our findings in Experiment 1, which tested younger adult participants only in a between-groups design and found that tRNS to VLPFC enhanced face memory performance (relative to sham; Figure 1B) and was not modulated by baseline ability.

## General Discussion

This study sought to determine whether, and how, high frequency tRNS to VLPFC modulates face memory abilities in typical younger and older adults. The findings demonstrate mixed results. In Experiment 1, we found that individuals who received active tRNS of the VLPFC showed significant improvements in face memory performance, while those who received sham stimulation did not. Similarly, while the active group and sham group did not differ from each other in their baseline face memory abilities, the active group outperformed the sham group following stimulation. Building on this finding, we conducted a second experiment to examine if a similar pattern of results would be found for a non-face memory task. In Experiment 2 active tRNS to VLPFC did not modulate non-face visual memory in younger adults. Finally, in Experiment 3, we sought to replicate the findings from Experiment 1 and examine whether participant age (younger versus older) influenced the outcome of stimulation effects on face memory. In Experiment 3, we also changed our stimulation factor from a between-group design (i.e., one group receiving active stimulation, one group receiving sham stimulation) to a within-group design (i.e., participants received active stimulation in one session, but sham stimulation in another – order was counterbalanced and testing was one week apart). Using this experimental design, we observed a significant *reduction* in face memory performance in active relative to sham tRNS conditions (i.e., the opposite pattern to Experiment 1). This reduction was linked to individual differences in baseline performance, with higher performing participants showing greater reductions in performance following stimulation (this effect remained when controlling for age and order of testing).

Our findings are in line with a number of recent investigations showing the sensitivity of transcranial electrical brain stimulation effects to subtle differences in experimental design and individual variation in participants. They speak to the importance of internal replication prior to publishing findings on changes in performance following transcranial electrical brain stimulation. The findings also call into question bold claims regarding the generalisability and reliability of transcranial electrical brain stimulation as a one-size fits all tool to modulate performance across a range of contexts without: (1) prior evidence verifying that stimulation effects maintain with variation in design (e.g., that effects maintain across between- and within-participant designs), (2) prior evidence verifying that stimulation effects maintain with variation in task (e.g., that the effect on one task in a single domain can be found on similar tasks in that domain), (3) prior evidence verifying that stimulation effects maintain with variation in participants (e.g., based on cohort factors like age, gender, ethnicity, typicality), and (4) prior evidence verifying that stimulation effects maintain with variation in setting (e.g., testing the effects of stimulation at different baseline states/locations for participants given the risk of state-dependent effects on stimulation outcome). All of the above are important factors to consider when verifying the reliability of effects of transcranial electrical brain stimulation before wider application.

More widely, the current findings highlight the danger of generalizing findings based on a single study within a young adult sample (as in Experiment 1) to a wider cohort without systematic investigation beforehand. This is particularly relevant in the domain of face processing, given between- and within-group variability in groups with atypical face processing (e.g., Duchaine and Nakayama, 2006b). Based on Experiment 1 alone we may have concluded that tRNS offered potential to improve face processing, however, the discrepancy between the results of Experiment 1 and Experiment 3 challenges the reliability of the findings and calls into question the potential of using tRNS targeted at VLPFC as a tool to boost face processing. Overall, our findings add to evidence suggesting that individual difference factors may influence the efficacy and direction of transcranial electrical brain stimulation effects, and highlight the importance of considering these carefully before using the technique as a tool in cognitive rehabilitation / neural enhancement (Krause and Cohen Kadosh, 2014; Fertonani and Miniussi, 2017). The findings highlight the importance of following up single study findings of changes in performance following transcranial electrical brain stimulation and establishing what parameters do/do not lead to modulation effects in a given domain, before: (i) assuming that they generalize across settings in a one-size fits all manner and (ii) assuming that effects are reliable.

Any future work examining the effects of tRNS on face memory could address some limitations of the current set of experiments. These include the lack of anatomical control site – while our findings indicate that VLPFC stimulation may modulate face memory performance (Experiment 1 and 3), performance was measured relative to sham stimulation only. This means that any interpretation on the anatomical locus of effect should be treated with caution. Further, while we did not find any effect on a non-face visual memory task in Experiment 2, the lack of a direct statistical comparison to performance on a face visual memory task in the same participants is a limitation impacting conclusions about task specificity of the stimulation.

It would also be interesting for future work to consider why higher performing participants would show greater reductions in face memory performance following stimulation in Experiment 3. One possibility is that this effect may result from high- and low-performing individuals relying on different networks for face recognition. These baseline differences in brain state may then interact with the effects of stimulation. Several studies using non-invasive brain stimulation have shown that baseline performance can predict the magnitude of change in performance following tES (e.g., Tseng et al., 2012, 2018; Hsu et al., 2014, 2016; Krause and Cohen Kadosh, 2014; Benwell et al., 2015; Fertonani and Miniussi, 2017; Penton et al., 2017). It has been suggested that this may be due to differential recruitment of brain networks/brain state in high and low performers (Tseng et al., 2012; Krause and Cohen Kadosh, 2014). Experiment 3 may be particularly sensitive to this possibility, since we tested both younger and older participants. It has been shown that VLPFC activity is greater in low-performing older adults compared to high-performers when performing memory-based tasks. Further, high-performers engage different brain networks than low-performers (Cabeza et al., 2002). That high-performing older adults recruit additional brain regions has often been interpreted in the context of compensation, whereby the reorganization of neurocognitive networks is used to compensate for deficiencies associated with typical aging (Cabeza et al., 2002). One possible explanation for our findings is that introducing additional excitability to the VLPFC may perturb these network dynamics in high performers. Consistent with this possibility, it is well known that brain stimulation effects can be state-dependent (Silvanto et al., 2008). This is relevant in the current context because some stimulation was conducted while participants were completing the task. It is therefore possible that differential effects in high- versus low-performers may also be linked to differences in state-dependent outcomes of stimulation effects (potentially based on different brain networks that are recruited). Future work is needed to clarify these possibilities.

In addition, it may be interesting for future work to consider how different phases of learning and memory may be influenced by stimulation (e.g., encoding versus retrieval). For example, studies using transcranial direct stimulation (tDCS) have suggested that approaches designed to suppress or enhance cortical excitability can exert differential effects on learning depending on when they are applied (Dockery et al., 2009; Renzi et al., 2015). One example of this is the work of Dockery et al. (2009) who suggest that for early stages of learning, stimulation techniques that support noise reduction of neuronal activity may be useful to aid learning and memory, while for later stages of learning, stimulation techniques that enhance the efficacy of active connections may be more useful to aid learning and memory. As one possible mechanism of action for tRNS is to enhance weak neuronal signals via stochastic resonance (Terney et al., 2008), it may be the case that the outcomes of tRNS on learning and remembering faces are influenced by when in time tRNS is applied.

A final further consideration of our results is the choice of stimulation parameters used. While prior work has shown that the use of bilateral electrode montages for tRNS stimulation can modulate performance in cognitive and perceptual domains (e.g., Cappelletti et al., 2013; Snowball et al., 2013; Joos et al., 2015; Romanska et al., 2015; Campana et al., 2016; Popescu et al., 2016; van der Groen and Wenderoth, 2016; van Koningsbruggen et al., 2016; Looi et al., 2017; Penton et al., 2017; Yang and Banissy, 2017), recent work using bilateral electrode montages for tRNS to stimulate the motor system has found that this approach does not induce the classical excitatory effects of unilateral tRNS (Parkin et al., 2018). This is similar to studies of transcranial direct current stimulation (tDCS: a more commonly used transcranial electrical brain stimulation technique), where many studies find behavioral differences using bilateral electrode montages, but work using bilateral tDCS montages to modulate activity in the motor system has found mixed results on motor cortex excitability changes (e.g., Nitsche and Paulus, 2000; Mordillo-Mateos et al., 2012; Sehm et al., 2013; Parkin et al., 2018). Similarly, while many studies show behavioral effects of tRNS using durations of stimulation for 10 min or more (e.g., Cappelletti et al., 2013; Snowball et al., 2013; Joos et al., 2015; Romanska et al., 2015; Campana et al., 2016; Popescu et al., 2016; van Koningsbruggen et al., 2016; van der Groen and Wenderoth, 2016; Looi et al., 2017; Penton et al., 2017; Yang and Banissy, 2017), recent work in the motor system suggest that excitatory effects of tRNS may not be present at longer durations of stimulation (comparing 10 to 20 min stimulation parameters; Parkin et al., 2018). One possible reason for this discrepancy is what happens in the motor system at rest may not necessarily predict what happens outside of it – e.g., studies using TMS have shown no correlation between motor system excitability and visual system excitability (e.g., Stewart et al., 2001; Antal et al., 2004). With that being said, given that the motor system is often used in transcranial electrical brain stimulation studies to infer neurophysiological effects it is important that we gain a better understanding of how different tRNS montages within and outside of the motor system influence cortical excitability before widely using the technique. This issue does not only relate to montages, but also the phase of stimulation (e.g., see Ho et al., 2015). All of the above leads to the conclusion that a greater consideration and understanding of the mechanisms of tRNS across different cohorts (e.g., young, old, typical, atypical) should be developed prior to wider use. Indeed, in a broader context, we would argue that a similar case should be made for the wide application of other transcranial electrical brain stimulation techniques (e.g., tDCS) across different cohorts where knowledge of the mechanisms of action/outcome is missing. This is not to conclude that the techniques should not be used in research studies, but that caution should be adopted before interpretation of findings and wide-scale use.

## Ethics Statement

The studies received ethical approval from Goldsmiths Research Ethics and Integrity Sub-Committee. All participants gave signed informed consent.

## Author Contributions

MB and TP were involved in all aspects of the experiments, including design, analysis, and interpretation. SB, KD, and BD contributed to study design and interpretation. MT, TR, MK, and SG contributed to data collection. All authors contributed to manuscript preparation.

## Conflict of Interest Statement

The authors declare that the research was conducted in the absence of any commercial or financial relationships that could be construed as a potential conflict of interest.
